# Multiple Myeloma-Derived Cellular Lipids Enhance Targeting and Efficacy of Nanoliposome Formulations In Vitro

**DOI:** 10.3390/ijms27188155

**Published:** 2026-09-13

**Authors:** Mathew Amorin, Christina Tran, Eden Park, Pedro L. Rodriguez Flores, William Smullen, Peter Daley, Kenny Pham, Shivmani Barve, Robert B. Campbell

**Affiliations:** 1 School of Pharmacy, Massachusetts College of Pharmacy and Health Sciences, 19 Foster Street, Worcester, MA 01608, USA; 2 Department of Pharmaceutical Sciences, Massachusetts College of Pharmacy and Health Sciences, 19 Foster Street, Worcester, MA 01608, USA

**Keywords:** cancer therapy, multiple myeloma, cell binding, drug targeting, liposomes, LDH, cytotoxicity, drug release

## Abstract

Multiple myeloma (MM) is a blood cancer characterized by the accumulation of abnormal plasma cells within the bone marrow (BM). The neoplastic expansion progressively disrupts normal blood cell production by outcompeting healthy hematopoietic cells. The disease is characterized by terminally differentiated B cells that secrete antibodies as part of an adaptive immune response. A serious threat confronting small drug molecule therapy for MM is the lack of selective drug targeting, ultimately resulting in insufficient accumulation of drugs to target cells, and harmful off-target drug effects. We now report on the use of cellular lipid extracts (LEs) originating from two different human multiple myeloma (target) cell lines, RPMI 8226 and NCI H929, to develop RPMI 8226 LE- and NCI H929 LE-modified nanoliposomes, respectively. Other ingredients included phospholipid DOPC (dioleoyl-phosphatidylcholine) and/or Chol (cholesterol). Additional cell lines included non-target (Y79-retinoblastoma, U937-lymphoma, K562-GFP-chronic myeloid leukemia) and (off-target) normal healthy PBMCs—peripheral blood mononuclear cells. The LE-modified nanoliposomes stably incorporated various cytotoxic agents, demonstrating novel formulation characteristics and cell-interaction profiles. RPMI 8226 LE enhanced nanoliposome targeting to source-originating RPMI 8226 cells, with diminished uptake by Y79 and PBMCs. The inclusion of Chol reduced targeting to RPMI 8226 cells. The RPMI 8226 LE enhanced nano-formulation effects against RPMI 8226 cells, but not against the Y79 control. Consistent with these findings, RPMI 8226 LE-modified nano-formulations enhanced extracellular lactate dehydrogenase release against RPMI 8226 cells, but not against the Y79 control. NCI H929 LE material also enhanced targeting and nano-formulation effects against source and non-originating source tumor cells from the BM. Designing nanoliposomes to mimic tumor cell membranes may represent a powerful strategy to treat multiple myeloma. Significance: Cellular membrane lipid extracts derived from multiple myeloma cells enhanced targeting and cytotoxicity while limiting damage to normal healthy cells. The inclusion of LE materials in drug delivery systems may represent a promising strategy to enhance cellular targeting and drug therapy for multiple myeloma.

## 1. Introduction

Multiple myeloma (MM) is an incurable plasma cell disorder that originates in the spongy bone marrow (BM). The disease is caused by irregular signaling, protein expression, and abnormalities involving dysfunctional plasma cells [1,2]. The renegade precursor B lymphocytes rapidly proliferate, eventually crowding out the population of healthy hematopoietic cells [3,4]. The disease affects older individuals. The average age of diagnosis is estimated at 69 years; for all SEER stages, the 5-year relative survival rate is 60% [5,6]. Diagnosed cases have largely progressed to advanced disease stages, resulting in a multitude of clinical challenges [3,7].

One drawback of employing cytotoxic drug agents for cancer therapy is insufficient drug transport, impeded by a number of tumor physiological barriers [8,9]. Nanoliposomes (aka~ liposomes) have been shown to address some of these challenges. Successes include enhanced drug targeting, improved drug pharmacokinetics, and modest benefits to patient survival [10,11,12,13]. Early preclinical and clinical outcomes continue to offer support for developing nanotherapeutics for the treatment of solid malignancies, and for the management of blood cancer diseases [4,14,15].

The chemical modifications and physical manipulations of conventional nanoparticles have assisted in disease management. These include alterations of particle size and surface charge characteristics. Modifications include the inclusion of cholesterol (Chol), synthetic polymers, antibodies, and specialized functional ligands [10,16,17,18,19,20]. However, research shows that cell membrane-mimicking nanoparticles improve drug targeting. They also increase therapeutic effectiveness. For example, the incorporation of total lipid isolates in the preparation of nanoparticles has enhanced the targeting of macrophages and breast cancer cells [21,22].

The studies conducted here support the use of tumor cell membrane-mimicking nanoliposomes to enhance targeting and nanoliposomal drug effects for MM. For the proof-of-concept study, two different LE materials derived from different human MM cell lines were evaluated throughout. The findings collectively support spontaneous, relatively selective and/or versatile lipid extract (LE)-mediated targeting. Cytotoxicity experiments were additionally employed to investigate the effectiveness of various LE-modified nano-formulations, combined with extracellular lactate dehydrogenase (LDH) metabolic activity and drug release studies. Furthermore, the application of the LE material protected healthy bone marrow cells while enhancing cytotoxicity against tumor cells that originated from the same microenvironment. Growing evidence positions LE-modified nanoliposomes as a promising strategy for the treatment of MM.

## 2. Results

### 2.1. RPMI 8226 LE Produced Actively Dividing Suspension and Senescent Adherent Cells in Mature Cell Cultures

**Figure 1:**
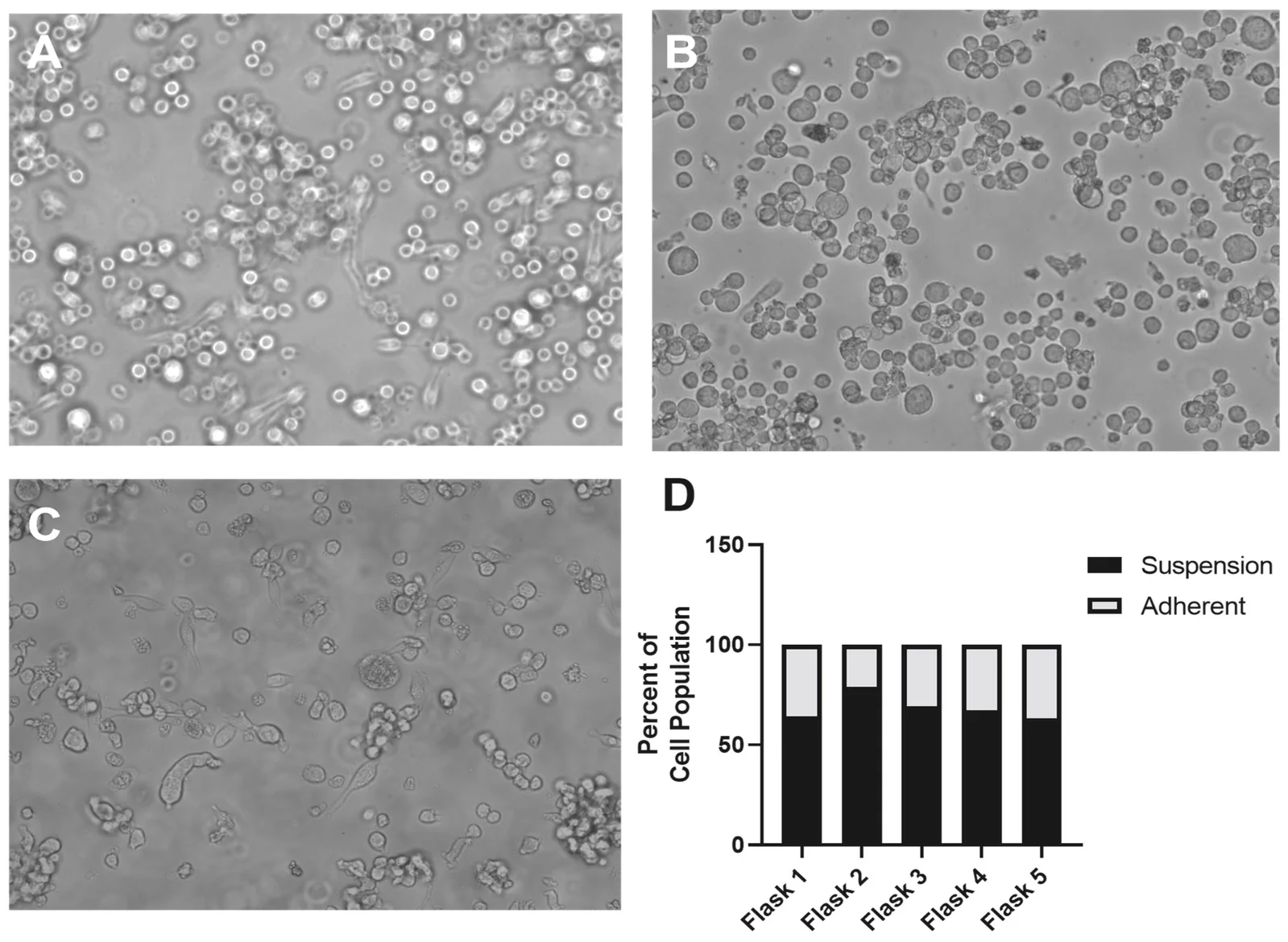
RPMI 8226 cellular growth characteristics. (**A**) An established RPMI 8226 cell population containing both suspension and adherent cell populations. (**B**) Adherent cells separated from suspension cells. To rid the cell suspensions of any cell debris the growth medium was removed, followed by a centrifugation wash step with sterile 1X PBS. The PBS was next replaced with fresh growth medium with cells returned to tissue culture flasks. After four weeks of undisturbed incubation the adherent cells became progressively senescent; no new suspension or adherent cells populated the flask. (**C**) The suspension cells removed from the original flask were centrifuged/washed with 1X PBS, and placed in a new flask; the population of cells gave rise to an established mix of suspension and adherent RPMI 8226 cells. (**D**) The distribution of suspension (floating) versus adherent (attached) cellular populations remained consistent across the five different tissue culture flasks. 20X magnification.

**Figure 2:**
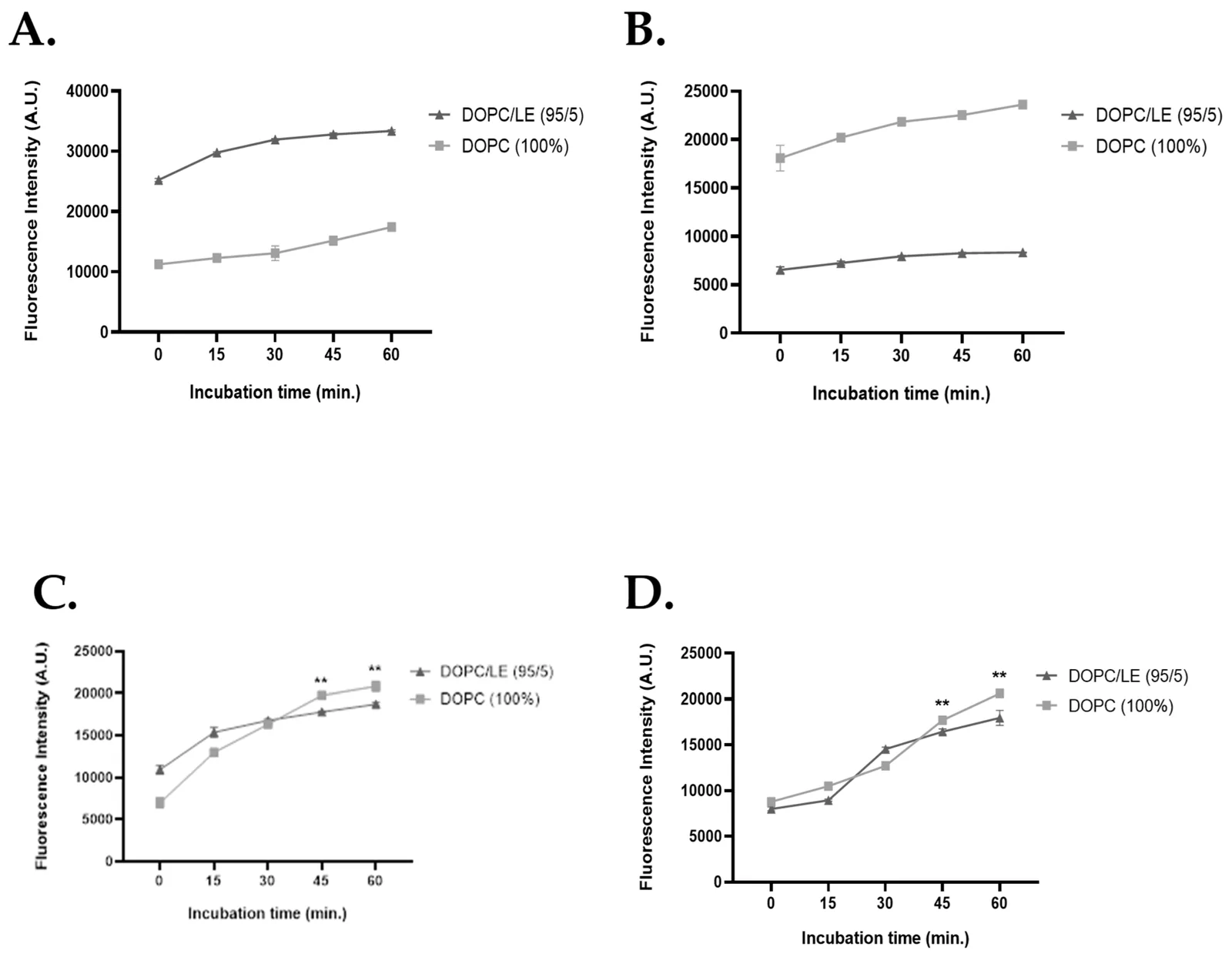
RPMI 8226-LE-modified nanoliposomes preferentially interact with target over non-target and off-target cell populations. Cells were exposed to (100 nmoles) rhodamine-labeled DOPC (100%) or rhodamine-labeled DOPC/LE (95/5) nanoliposomes (*n* = 4) per ml of growth medium. Cell suspensions incubated with nanoliposome preparations (between 0 and 60 min) with the oscillator rate set on low in a humidified chamber (see Section 4). Following a centrifugation and PBS wash step to separate cellular bound from unbound nanoliposomes, fluorescence intensity was used to determine the cellular bound fraction of nanoliposomes at different time intervals. Data represent combined results from multiple different experiments. (**A**) Panel A shows greater binding and uptake of DOPC/LE nanoliposomes by (target) RPMI 8226 cells compared to DOPC nanoliposomes. (**B**) Panel B shows reduced binding/uptake of DOPC/LE nanoliposomes by (non-target) Y79 cells compared to DOPC nanoliposomes. (**C**) The U937 (non-target) cells were exposed to DOPC and DOPC/LE nanoliposome preparations. (**D**) The PBMCs (off-target) cells were exposed to DOPC and DOPC/LE nanoliposomes. Data collectively support selective targeting characteristics of RPMI 8226 LE-modified nanoliposomes compared to conventional controls in vitro. ** *p* < 0.01.

The successful isolation of total lipid extracts from MM cells depends heavily on the growth profile of the specific cell line. First, once target cells reached 50–80% confluency in T-75 tissue culture flasks, they were transferred to T-225 flasks for expansion. The primary cultures became populated with healthy suspension (floating) cells. Mature RPMI 8226 cell cultures contained a mix of single cells and clusters, unexpectedly consisting of floating and adherent cells (Figure 1A). The adherent cells were multicellular aggregates dispersed irregularly throughout the flask (Figure 2B).

The removal of suspension cells from the mature culture revealed an undisturbed population of relatively slow-growing adherent cells that became progressively senescent, failing to produce additional floating or adherent cells. The prevalence and role of cellular senescence in hematological malignancies has been documented [23]. However, the separated population of suspension cells produced adherent and floating cells (Figure 1C). 

To quantify the ratio of suspension to adherent cells in a mature cell culture, five flasks of RPMI 8226 cells were grown to approximately 80% confluency. A cell population density of 68 ± 6% and 32 ± 6% was observed for viable suspension and adherent cells, respectively (Figure 1D). For clinically relevant purposes mixed populations of floating and adherent cells were used for the lipid extraction.

### 2.2. Optimized Physicochemical Characteristics of RPMI 8226 LE-Modified Nanoliposomes

Molecular charge and size of nanoparticles are important determinants of targeting. The physical characteristics have been shown to influence the residence time of drug delivery vehicles in blood and the extent to which they are internalized by target cells, and can limit uptake by healthy organ tissues [24,25]. The size of the nanoliposomes utilized in this study ranged between 175 and 311 nm, with a collective mean diameter of 259 ± 50 nm (Table 1). Sonication significantly reduced general particle sizes for LE-modified and control nanoliposomes, producing more uniform populations with time. The measurements for all sonicated drug-loaded nanoliposomes revealed two different effects on molecular size. Firstly, the incorporation of doxorubicin and carboplatin in DOPC (100%) produced smaller nanoliposomes when compared to drug-loaded LE-modified varieties (Table 2). Secondly, the formulations containing doxorubicin were the largest of the preparation types evaluated (>200 nm; PDI ~0.2). Mean diameters for carboplatin and gemcitabine were smaller on average (ranging between 88 and 112 nm; PDI between 0.1 and 0.2). Zeta potential values for the LE-modified nanoliposomes generally ranged from neutral to slightly more negative than the drug-loaded DOPC control (Table 2). 

Overall, the effect of RPMI 8226 LE on nanoliposome size was inconsistent, producing both smaller and larger particles when compared to the drug-loaded DOPC controls.

### 2.3. RPMI 8226 LE Enhanced Nanoliposome Targeting, but the Additional Inclusion of Cholesterol Diminished the Uptake

The inclusion of breast cancer lipid extract (4T1-LE) and cholesterol in nanoliposomes revealed beneficial formulation and cellular properties in vitro [21]. To optimize targeting, the next experiment investigated how cholesterol content affects RPMI 8226-modified nanoliposome binding and uptake by source-originating MM cells.

The data show that the inclusion of cholesterol significantly increased the uptake of DOPC (100%) nanoliposomes by RPMI 8226 cells (*p* < 0.001). The inclusion of RPMI 8226 LE also improved the cellular uptake of nanoliposomes by RPMI 8226 cells (*p* < 0.001). Interestingly, nanoliposomes consisting of DOPC/LE alone targeted RPMI 8226 cells to a greater extent compared to DOPC/cholesterol/LE (90/5/5; *p* < 0.001), with reported fluorescence intensity values of 29,008 (*n* = 4) and 26,572 (*n* = 4), respectively (Table 1). The additional inclusion of cholesterol (5 mol%) increased the uptake of nanoliposomes consisting of DOPC (100%). However, cholesterol decreased the uptake of LE-modified nanoliposomes. The optimized composition/ratio for targeting RPMI 8226 cells was DOPC/LE (95/5). 

### 2.4. Spontaneous, Selective and Differential Uptake of RPMI 8226 LE-Modified Nanoliposomes 

RPMI 8226 LE-modified nanoliposomes may be employed to target multiple myeloma cells. However, as shown in the cholesterol study, the extent of targeting is composition- and ratio-dependent. Understanding the conditions to achieve differential and selective cellular uptake is critically important for optimization and applications. 

In this next study, suspensions of target (RPMI 8226), non-target (Y79 and U937) and off-target PBMCs (peripheral blood mononuclear cells) were seeded at 1 × 10^4^ cells per ml of growth medium in centrifuge tubes. RPMI 8226 cells were first exposed to fluorescently labeled DOPC (100%) or DOPC/LE (95/5) nanoliposomes, followed by incubation. Data show lower binding of DOPC nanoliposomes to RPMI 8226 cells compared to DOPC/LE nanoliposomes regardless of the time of incubation (Figure 2A). In fact, differential cell uptake occurred without incubation, as observed at the 0-min time point. RPMI 8226 LE-mediated cell targeting was spontaneous and selective (Figure 2A).

Additional studies investigated the influence of the cellular lipid extract on nanoliposome uptake by non-target (Y79 and U937) cells. Cellular binding and uptake of DOPC (100%) by non-target Y79 cells was greater compared to LE-modified nanoliposomes at all incubation time points evaluated (Figure 2B). However, enhanced uptake of DOPC (100%) nanoliposomes by U937 cells was observed when compared to DOPC/LE (95/5) between 45 and 60 min of incubation (*p* < 0.001; Figure 2C). 

Off-target drug effects include a complex host of toxicities, often resulting in irreversible damage to normal tissue [26,27,28]. Therefore, the next experiment assessed the relative uptake of LE-modified nanoliposomes by normal, healthy (off-target) cells, using peripheral blood mononuclear cells (PBMCs) as the non-originating cell population. PBMCs served as the population of the off-target or non-originating source cells. Similar to experiments reported here for U937 non-target cells, the inclusion of RPMI 8226 LE in nanoliposomes reduced vehicle uptake by PBMCs between 45 and 60 min, compared to the DOPC control (*p* < 0.001; Figure 2D). The findings support spontaneous and selective uptake of RPMI 8226 LE-modified nanoliposomes by target cells, with reduced uptake by non-target and off-target cell populations.

### 2.5. RPMI 8226 LE Enhanced Cytotoxicity and Extracellular LDH Release 

The RPMI 8226 LE enhanced cellular targeting. The next step was to evaluate relative levels of (target and non-target) cells to the effects of drug-loaded RPMI 8226 LE-modified nanoliposomes. In this study, RPMI 8226 (target) and Y79 (non-target) cells were exposed to three cytotoxic drug agents, each loaded in nanoliposomes w/wo the inclusion of RPMI 8226 LE.

**Figure 3:**
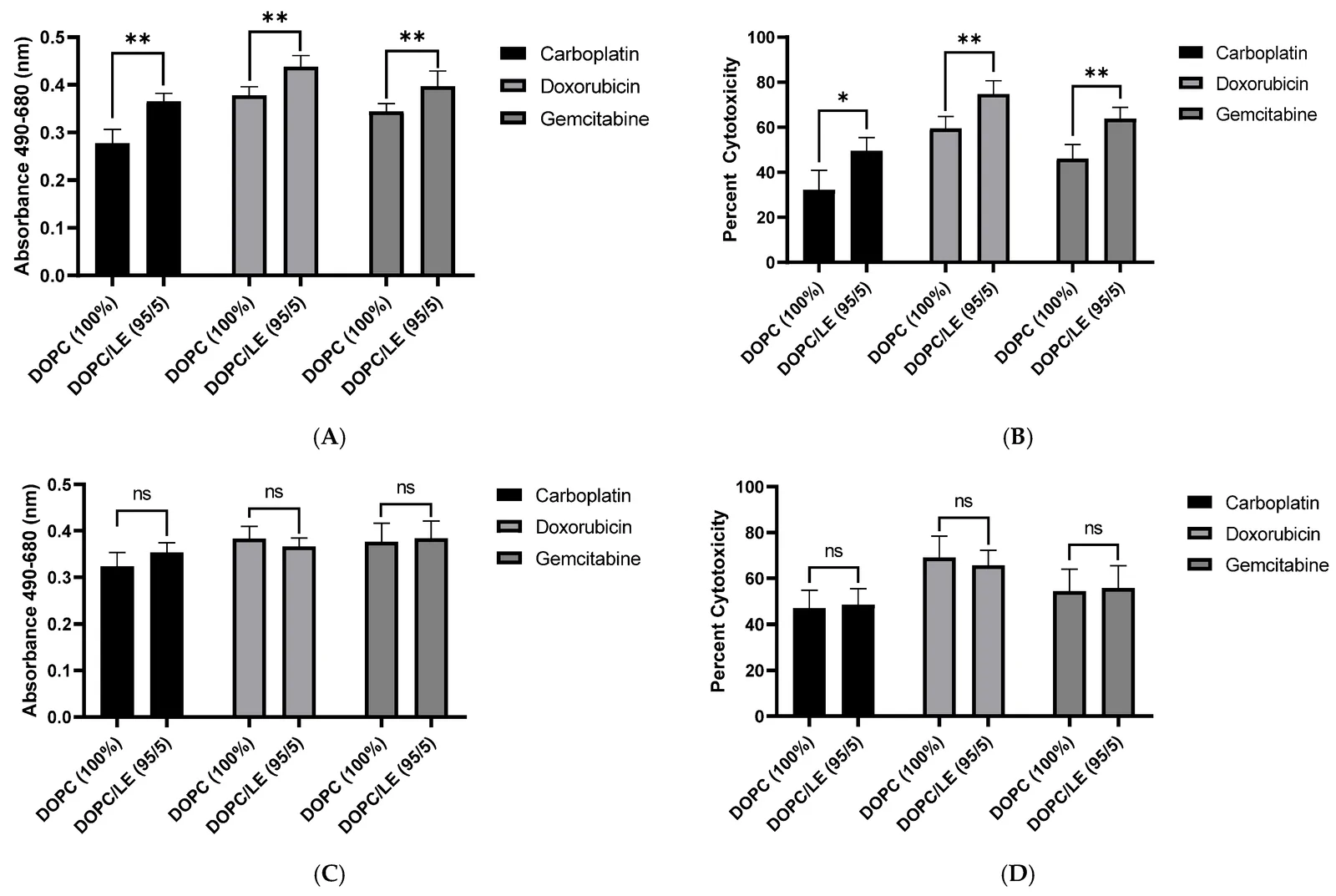
Extracellular LDH activity and cytotoxicity of formulations against multiple myeloma target versus non-target cells. Extracellular LDH activity was measured by red formazan absorbance at 490 and 680 nm following RPMI 8226 MM cell exposure to (**A**) DOPC (100%) or DOPC/LE (95/5) loaded with (3 mol%) drug. See higher LDH activity levels for (DOPC/LE) compared to DOPC control. (**B**) The LDH activity of chemically treated, untreated, and fully lysed RPMI 8226 cells are shown. Graph shows cytotoxicity for drugs loaded in RPMI 8226 LE preparations and DOPC controls. (**C**) Graph shows extracellular LDH levels detected for (negative control- non-target) Y79 cells following exposure to DOPC (100%) or DOPC/LE (95/5) preparations. (**D**) Graph shows percent cytotoxicity of control Y79 cells with drug-loaded preparations composed of DOPC (100%) or DOPC/LE (95/5). Data show that the RPMI 8662 LE material enhanced nano-formulation effects against MM. Data show results from 3 different experiments. Note: ns = not significant, * *p* < 0.05, ** *p* < 0.01.

Given that LDH assays measure LDH released from treated MM cells, we measured extracellular LDH levels and compared them with the cytotoxicity profiles. The data revealed that all three RPMI 8226 LE-modified nanoliposomal formulations effectively managed the growth of RPMI 8226 cells. The observation was further supported by significant extracellular LDH profiles for all three drug agents when loaded in the DOPC/LE (95/5) nanoliposomes, compared to the corresponding DOPC (100%) nanoliposome controls (*p* < 0.01; Figure 3A,B). Moreover, there was no significant difference in extracellular LDH release or cytotoxicity when comparing LE-modified RPMI 8226 nanoliposomes to control nanoliposomes against Y79 cells (*p* > 0.05; Figure 3C,D). In summary, the inclusion of RPMI 8226 LE enhanced the effectiveness of nanoliposome formulations against RPMI 8226 cells, but provided no benefit when employed against non-originating source tumor cells. The findings collectively support that drug-loaded RPMI 8226 LE nanoliposomes are relatively selective, enhancing cytotoxicity against target more than non-target cells.

### 2.6. LE-Modified Nanoliposomal Therapeutics Diminished Cytotoxicity Against Normal Healthy BM Cells

Off-target cytotoxicity is a frequent result of drug therapy that may cause irreversible damage to normal tissue [27]. Nanotherapeutics maximize the delivery of drugs to target cells, while limiting the potential harm to normal healthy cells. Whether the additional inclusion of RPMI 8226 LE material will increase or decrease off-target effects is not yet known. To evaluate off-target toxicity, PBMCs isolated from healthy donors were used as a cellular model for normal tissues.

**Figure 4:**
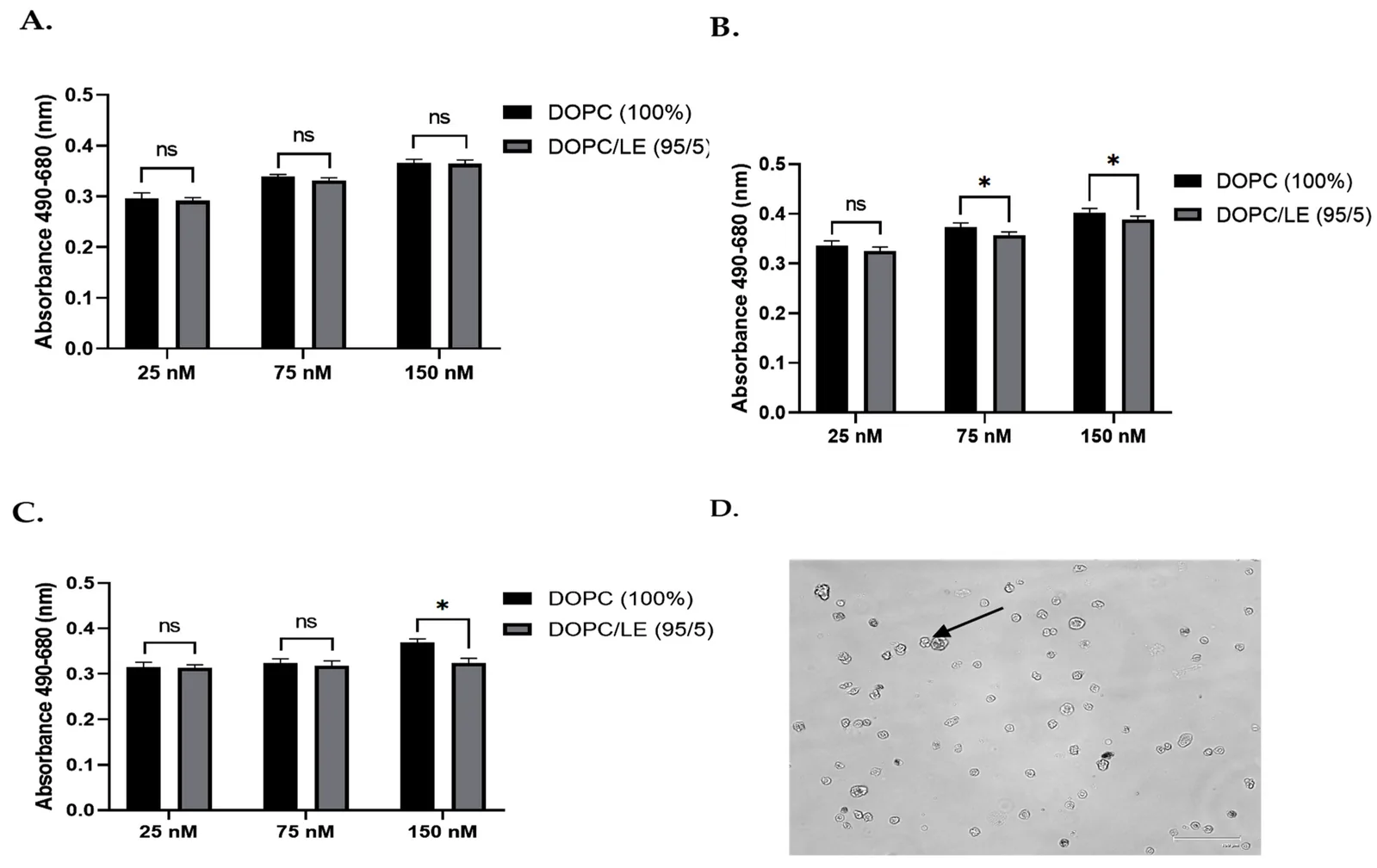
Extracellular LDH activity: Comparison of formulation effects against normal peripheral blood mononuclear cells. Extracellular LDH activity was measured and evaluated following overnight exposure of PBMCs to drug-loaded DOPC (100%) and DOPC/LE (95/5) preparations containing (3 mol%) drug agent. (**A**) DOPC (100%) and DOPC/LE (95/5) preparations loaded with carboplatin. (**B**) DOPC (100%) and DOPC/LE (95/5) preparations loaded with doxorubicin. (**C**) DOPC (100%) and DOPC/LE (95/5) preparations loaded with gemcitabine. (**D**) Image depicts PBMCs maintained in a T-150 flask. Healthy population of PBMCs consisted of mixed composition of adherent and suspension cells in single and cluster cell populations. Data represent results from 3 different experimental determinations. 20X magnification. Scale bar = 100 µm. (**D**) Arrow (↓) shows a single cluster of cells in suspension. Note: ns = not significant, * *p* < 0.05.

Single cell and multiple cell clusters of PBMCs (Figure 4) were exposed to LE-modified nanoliposome therapeutics. Cytotoxic drug agents (carboplatin, doxorubicin and gemcitabine) were loaded in DOPC (100%) and DOPC/LE (95/5) nanoliposomes. The PBMCs were exposed to drug-loaded RPMI 8226-LE and corresponding control preparations. Detection/analysis of LDH and cytotoxicity followed a 24 h incubation period. 

The exposure of PBMCs to carboplatin loaded in nanoliposomes and in RPMI 8226 LE-modified nanoliposomes resulted in similar extracellular LDH release profiles (Figure 4A). An elevated extracellular LDH level was observed for doxorubicin-loaded DOPC when compared to the LE-modified nanoliposome variety (*p* < 0.05; Figure 4B). Similarly, the gemcitabine-loaded DOPC (100%) nanoliposomes exhibited greater extracellular LDH release compared to the LE-modified nanoliposomes (*p* < 0.05; Figure 4C). In summary, the inclusion of RPMI 8226 LE either resulted in similar LDH levels to the control nanoliposomal preparations or demonstrated reduced off-target cytotoxicity. The RPMI 8226 LE-modified nanoliposomes preferentially accumulated in the target over other cell classifications. Although the LE-modified nano-formulations did not increase cytotoxicity against healthy cells, the profile of these systems may depend on the specific encapsulated drug. This drug-dependent effect is evidenced by the similar extracellular LDH release profiles observed between DOPC and LE-modified nanoliposomes for carboplatin.

### 2.7. NCI H929-LE Nanoliposomes Preferentially Target Tumor Cells Originating from the BM

**Figure 5:**
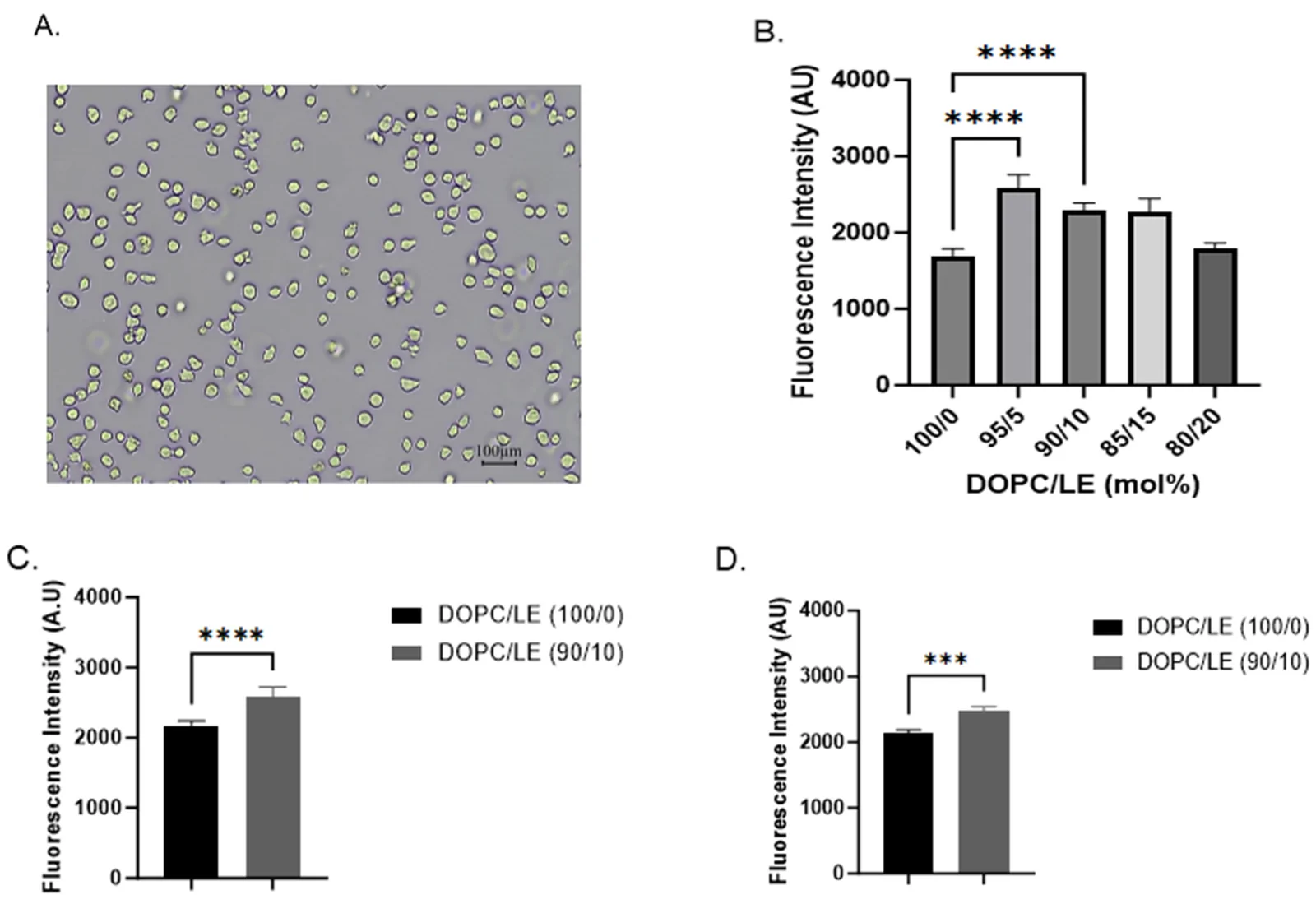

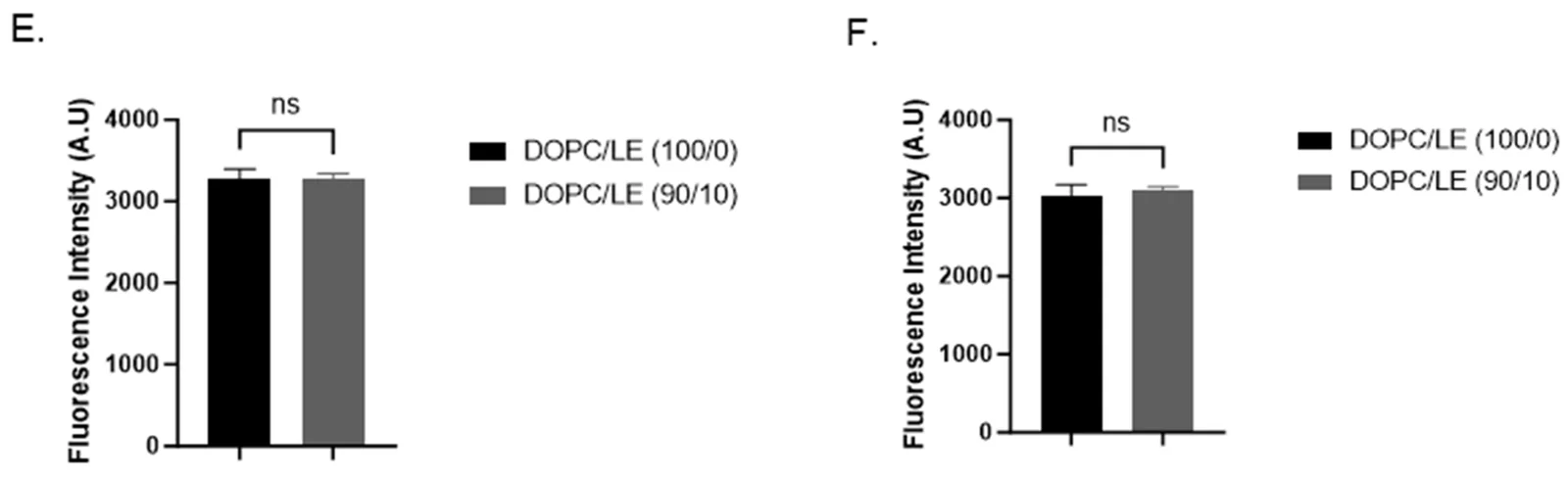
NCI H929-modified nanoliposomes preferentially target BM cancer cells. Cell suspensions were incubated with rhodamine-PE fluorescently labeled nanoliposomes for 1 h with the oscillator set on low in a humidified chamber. Following centrifugation to separate cellular bound versus unbound nanoliposomes, the nanoliposome-bound cell fraction was transferred to 24-well plates, and analyzed using a microplate reader. Fluorescence intensity values were used to assess cellular uptake. (**A**) NCI H929 (target), (**B**) NCI-H929 (target), (**C**) U937 (non-target), (**D**) K562-GFP (non-target), (**E**) Y79 (non-target), and (**F**) PBMC (off-target) cell populations. Transformed BM cells were preferentially targeted over cells derived from other tissue environments. Note: ns = not significant, *** *p* < 0.001, **** *p* < 0.0001.

The RPMI 8226 LE studies employed LE material derived from a single MM (RPMI 8226) cell line. It is not known whether the beneficial properties reported here for RPMI 8226 and the corresponding LE material can be repeated with the use of a different MM cell line, and LE. Therefore, we employed the MM cell line, NCI H929. Following expansion of the fully adherent cell line in tissue culture flasks, followed by cellular lipid extraction of NCI H929 cells, we evaluated the cellular targeting characteristics of the NCI H929 LE-modified nanoliposome and unmodified nanoliposome control. To study the influence of NCI H929 LE on targeting, NCI H929 cells (shown in Figure 5A) were exposed to NCI H929 LE-modified and unmodified nanoliposomes. In this experiment, NCI H929 LE (0 to 20 mol%) improved the uptake by NCI H929 (target) cells. 

An amount of 5 to 15 mol% NCI H929 LE enhanced targeting compared to the unmodified nanoliposome control (*p* < 0.0001; Figure 5B). No significant difference in cellular uptake was observed between nanoliposomes containing 0 and 20 mol% of NCI H929 LE (*p* > 0.05). To understand how the NCI H929 LE material affects future cellular targeting and formulation studies, an amount of 10 mol% of LE material was used in the subsequent experiments. 

The inclusion of (10 mol%) NCI H929 LE was shown to enhance nanoliposome uptake by non-target cells. This was also observed with lymphoma-U937 (*p* < 0.0001) and CML-K562-GFP (*p* < 0.0001) cell lines, as shown in Figure 5C and Figure 5D, respectively. Interestingly, the beneficial effect of NCI H929 LE on the non-target cells did not include the retinoblastoma Y79 cells (*p* > 0.05; Figure 5E). There was also no difference in the uptake of NCI H929 LE-modified and unmodified nanoliposomes by the PBMCs (Figure 5F). 

NCI H929 LE enhanced nanoliposome uptake by target and non-target tumor cells (NCI H929, U937, K562-GFP) originating from the BM. However, the enhanced uptake was not observed with the Y79 cells. Of note, the retinoblastoma (Y79) cell line naturally develops in the retina. This represents an anatomically different environment from the BM. Moreover, while the NCI H929 LE enhanced nanoliposome uptake by BM tumor cells, the enhanced uptake did not include the normal healthy cells from the BM.

### 2.8. NCI H929 LE Improved Nano-Formulation Drug Effects Against Tumor Cells Originating in the Bone Marrow

**Figure 6:**
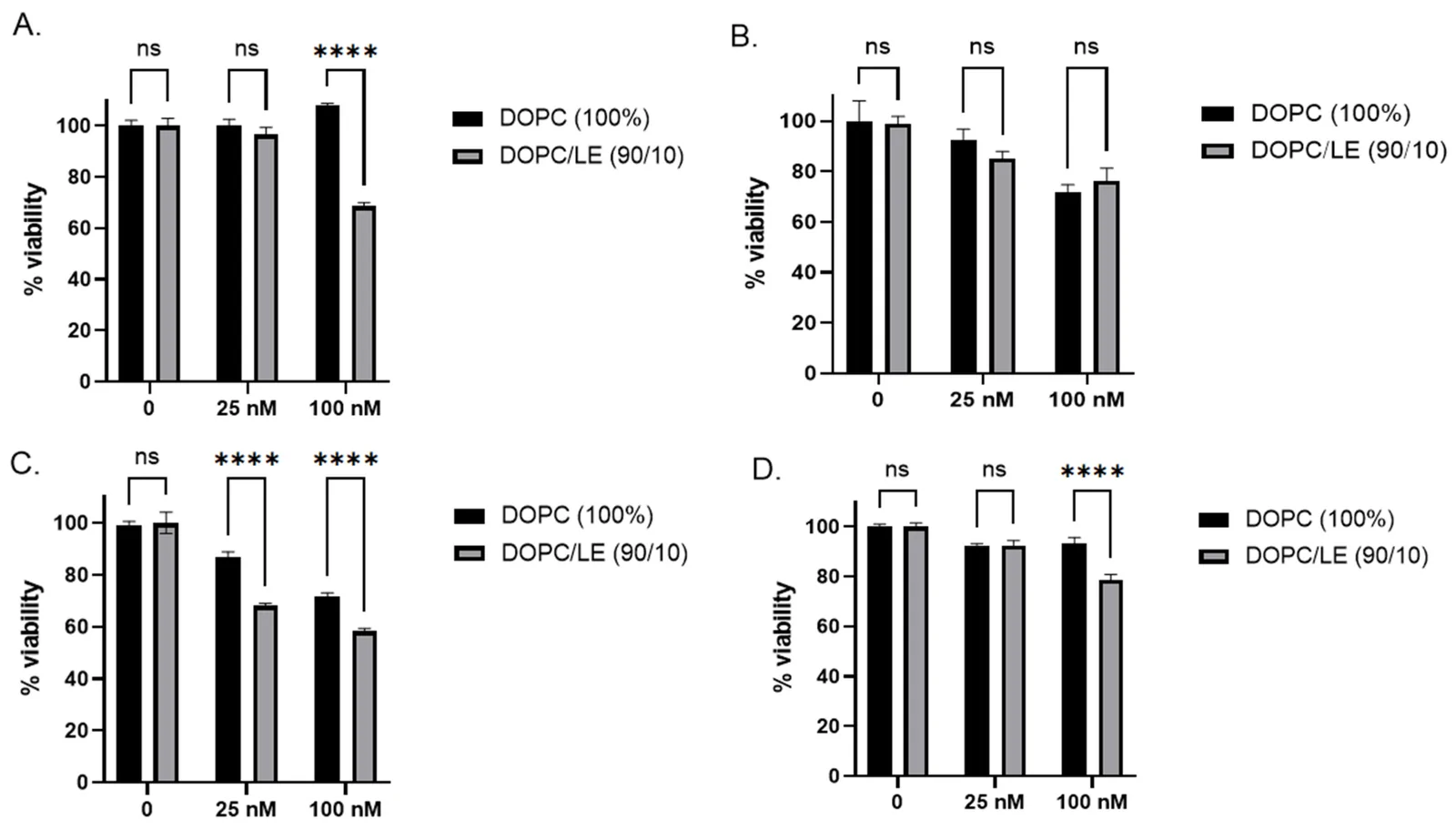
Nano-formulation Effects: Evaluation of NCI H929 LE-modified versus unmodified formulations. Graphs show the effect of including NCI H929 LE in nanoliposome preparations compared to nanoliposome control against target (NCI-H929) and non-target (U937) cells. Graphs shown in panels A&B: (**A**) NCI H929 and (**B**) U937 represent the results of cellular exposure to nanoliposomes loaded with doxorubicin. Graphs shown in panels C&D: (**C**) NCI-H929 and (**D**) U937 represent results of cellular exposure to nanoliposomes loaded with bortezomib. Data are represented as % viability of control. Transformed BM cells were susceptible to NCI-H929 LE-modified nano-formulations. The application of cytotoxic drug-loaded NCI-H929 LE-modified nanoliposomes was drug-dependent. Note: ns = not significant, **** *p* < 0.0001.

The next study investigated whether these enhanced cellular interactions translated to improved drug efficacy. For this reason, two different cytotoxic drug agents were loaded into NCI H929 LE-modified and unmodified nanoliposomes. The first agent was doxorubicin, evaluated in the RPMI 8226 LE study. The second agent was bortezomib (Velcade); the proteasome inhibitor is clinically approved for the treatment of MM and Mantle Cell Lymphoma. Formulation effectiveness was evaluated using the SRB cytotoxicity assay. The NCI H929 and U937 cell lines used represented the intended target and non-target cells, respectively. Each cell type was exposed to LE-modified and unmodified nano-formulation drug controls (Figure 6). 

NCI H929 cells were more susceptible to the effects of doxorubicin-loaded NCI H929 LE-modified nanoliposomes, compared to the unmodified variety (*p* < 0.0001). There was no difference in the growth-inhibitory effects between the two formulation types when employed against the U937 cells (*p* > 0.05). Similarly, bortezomib-loaded NCI H929 LE-modified nanoliposomes demonstrated greater cellular growth inhibition against NCI H929 cells compared to the corresponding control (*p* < 0.0001). Interestingly, bortezomib loaded into LE-modified nanoliposomes demonstrated greater growth-inhibitory effects against U937 cells (at 100 nM) than when delivered via the unmodified DOPC control (*p* < 0.0001). 

The inclusion of NCI H929 LE in nanoliposomes improved cellular uptake and nano-formulation effects against the NCI H929 cells. The observed targeting characteristics of the NCI H929 LE material were further supported by the cytotoxicity experiments. Furthermore, NCI H929 LE nanoliposomes showed a broader affinity for bone marrow tumor cells than the RPMI 8226 LE-mediated nanoliposomes.

### 2.9. RPMI 8226 and NCI H929 LE-Modified Formulation Drug Release

The amount of drug released from nanoparticles is a key predictor of drug performance, directly linking formulation stability to efficacy and safety. We therefore determined whether LE-modified nanoliposomes exhibited a rapid burst release or an extended release of drug payloads, and compared the LE-modified formulations to the unmodified controls.

In this study, the percent of drug released for select doxorubicin (Dox) and bortezomib (BTZ)-loaded RPMI 8226 LE and NCI H929 LE-modified nanoliposomes was compared to the respective drug-loaded DOPC nanoliposome controls. To replicate the conditions of the cytotoxicity experiments, all formulations were incubated at 37 °C in an FBS-supplemented cell culture medium. 

Overall, the initial encapsulation efficiency (%EE) for the drug-loaded nanoliposomes ranged from 90 to 98% (Table 3). When subtracted from the baseline %EE values reported in Table 3, the total amount of Dox and BTX released from the nanoparticles was under 10%. DOX was also released from LE-modified nanoliposomes in greater amounts than from the DOPC control at 72 h (Table 3). Conversely, about 4 to 5% of BTZ was released from RPMI 8226-LE-modified nanoliposomes and NCI H929-LE nanoliposomes, compared to 7% for the DOPC control (Table 3).

The findings align with previous research demonstrating that LE material may be employed to successfully load chemotherapeutic agents with efficiency comparable to or exceeding conventional controls [21]. With more than 90% of BTZ and Dox incorporated in the nanoliposomes under simulated physiological conditions after 72 h, LE-modified nanoliposomes may retain therapeutics and effectively deliver their drug payloads.

## 3. Discussion

The use of antibody conjugates, specialized ligands, and novel synthetic lipids has improved the development of nanomedicines worldwide [10,11,16,19]. Cell-derived lipids have inspired early next-stage efforts in the development of vaccine adjuvants and cancer nanotherapeutics [21,22]. The main objective of this study was to investigate the effects of including multiple myeloma cell membrane-derived LE material in the preparation of nanoliposomes. The cell lines employed were designated as (i) target, (ii) non-target, and (iii) off-target populations. 

The two *target* or originating-source cell lines (RPMI-8226 and NCI-H929) represented the original source from which the lipid extract (LE) was derived, serving both as the exclusive source of LE material and the primary target for LE-modified nanoliposome interaction. The *non-target* or non-originating source cell lines (Y79 retinoblastoma and U937 lymphoma) were not used for LE extraction but rather served to evaluate nanoliposome selectivity. Finally, the off-*target* PBMCs served as a model for the healthy human tissues typically subjected to unintended cytotoxicity during multiple myeloma therapy.

The cultured RPMI 8226 cells consisted of a population of floating and adherent cells. The floating (suspension) fraction produced both adherent and additional floating cells. The adherent cells became senescent with limited capacity for growth, while the floating population continued to proliferate, expanding to eventually consist of a mixed cell population (Figure 1). The distribution of approximately 2/3rd to 1/3rd of suspension to adherent cells remained relatively consistent among the flasks (Figure 1D). Regardless of the cellular senescence of the adherent cell population, the decision was made to include the mixed cell population in the lipid extraction process, thus aligning with efforts to obtain the most effective LE material. Of note, tumor cells are also supported by cellular senescence in the BM, playing a key role in the regenerative activity of mature cancer cells [23].

Molecular size and charge are relevant physicochemical properties affecting nanoparticle entry into cells. For instance, molecular cut-off size and charge influence tumor vascular and interstitial diffusional transport [24,29,30]. Additionally, nanoliposome surface charge alters interactions with circulating blood proteins. In this study, the hydrodynamic diameters of all drug-loaded nanoliposomal formulations, including those utilized in the drug release study, ranged from 90 to about 300 nm. Among these, the carboplatin- and gemcitabine-loaded formulations exhibited the smallest sizes, ranging between 90 and 112 nm. The zeta potential values for the drug-loaded, LE-modified nanoliposome formulations were slightly lower (more negative) than those of the unmodified varieties (Table 2). Throughout the study the LE-modified nanoliposomes demonstrated sensitivity to both the type and duration of sonication, consistently yielding hydrodynamic sizes under 200 nm. Like most physical sizing methods, the reported values do not represent the absolute minimum dimensions; instead, they reflect the specific characteristics of nanoliposomes generated under consistent conditions for these experiments. Nonetheless, the values for nanoliposome size and charge were consistent with preparations documented elsewhere to improve drug targeting and effects [24,29,30].

The additional inclusion of cholesterol in the RPMI 8226 LE-modified nanoliposomes reduced their uptake by RPMI 8226 cells. There is enormous support for including cholesterol in the development of nanotherapeutics. Reasons include cholesterol’s influence on drug incorporation, retention, stability, membrane fluidity, permeability, and phospholipid packing in membranes [17,18]. With the benefits reported for including cholesterol in nanoliposome development, the reduced uptake observed for RPMI 8226 LE-modified nanoliposomes by target cells following the inclusion of cholesterol was not expected. Particularly since cholesterol was previously shown to enhance the cellular entry of different lipid extract-modified nanoliposomes by breast cancer cells [21], LE-modified nanoliposomes should thus be customized for their intended purpose. 

The influence of RPMI 8226 LE on the uptake of nanoliposomes by target cells should be characterized as both spontaneous and relatively selective. Spontaneous, given the favorable binding events that occurred with the LE-mediated nanoliposomes in the absence of oscillation/incubation (0 min. time pt.). The process is selective; DOPC nanoliposomes were taken up by non-target Y79 cells to a much greater extent compared to the LE-modified nanoliposomes (Figure 2B).

To date, the exact mechanism(s) underlying LE-mediated targeting are not well understood. As such, beneficial cellular interactions occur when the LE material derived from the target cells is employed against the same cell line. Therefore, the reduced uptake by non-target and off-target cells was not surprising. On the other hand, the similar cell uptake profiles observed for non-target U937 and off-target PBMCs (Figure 2C,D) warrant further discussion. For starters, both of these leukocyte cell lines originate from the bone marrow. In contrast, cellular uptake in the retina-derived Y79 cells was substantially lower than in the leukocyte lines. Taken together, these results suggest that LE-mediated nanoliposome targeting may be regulated, in part, by the tissue organ of the source cells. 

The composition of total lipids derived from cellular models of tumors versus normal healthy tissue has been studied [17,31]. To develop more selective nanotherapeutics, we aimed to prepare nanoliposomes with membrane characteristics that mimic those of the target cell population. Because RPMI 8226 cells encompass the total lipid fraction of both adherent and suspension cell populations, our LE-modified nanoliposomes share the same lipid composition and profile as the target cells. Consequently, the enhanced targeting of the LE material over control nanoliposomes likely stems from the membrane composition similarities between the drug carrier molecule and the target cell.

LDH (lactate dehydrogenase) expression has been linked to metabolic and immune suppression functions in hematological diseases including multiple myeloma [32,33,34]. The revised ISS (international staging system) for MM now includes the levels of lactate dehydrogenase in diagnosed patients as prognostic information [6]. Since extracellular LDH release is a reliable indicator of cellular health, levels of extracellular LDH were used to confirm alterations in cell health in response to nano-formulation effects. 

Collectively, these studies demonstrate enhanced extracellular LDH release and cytotoxicity for all drug-loaded, RPMI-8226 LE-mediated nanoliposomes compared to conventional controls (Figure 3A). Moreover, PBMC survival following exposure to the cytotoxic agents (doxorubicin or gemcitabine) was significantly higher when encapsulated in DOPC/LE nanoliposomes than in DOPC-only formulations. Cellular binding studies support this observation, suggesting that the protective effect on PBMCs is mediated by the incorporation of LE into the nanoliposomal bilayer.

As noted, cholesterol decreased the overall uptake of LE-modified nanoliposomes by RPMI 8226 cells. For this reason, the sterol compound was not included in the remaining nanoliposome preparations, including the evaluation of any other LE material. Employing the established PC-based phospholipid (i.e., DOPC)- both in the presence and absence of the lipid extracts allowed up to isolate the effects of the extract materials on nanoliposome cell behavior without outside interference.

We needed to confirm whether all MM lipid extracts possess similar characteristics, regardless of their source cell line. For the experiments involving the NCI H929 LE, the findings support an alternate use of employing cellular lipids for targeting. Given a particular disease (i.e., MM), the degree of target selectivity of the LE material may well depend on the MM cell line from which the LE was derived. For example, the RPMI 8226 LE produced nanoliposomes that were relatively selective, targeting the original source cell line more compared to others evaluated, including other transformed cells of the BM. Interestingly, although NCI H929 cells were the preferential target of LE-modified nanoliposomes, targeting was influenced by cell type and environment. This raises the question if a single cellular LE material would be effective against an array of related diseases.

Comprehensive studies investigating the underlying mechanism(s) regulating the entry of LE-modified nanoliposomes into target cells are warranted. MM cells depend on clathrin- and caveolin-dependent endocytosis and micropinocytosis to internalize lipids and extracellular vesicles [35,36,37]. The LE materials employed in this study enhanced cellular targeting and efficacy. However, the particular pathways for the internalization of the LE-modified nanoliposomes are unclear. With that said, we offer some possible explanations that warrant future consideration. First, phosphatidylcholine-rich nanoliposomes are internalized via endocytosis, and the cellular entry of nanoliposomes when prepared with polyunsaturated lipids is a rapid process [38,39,40,41]. Given that a relatively abundant PC content was combined with the RPMI 8226 LE and NCI H929 LE materials, endocytosis may represent a major route of cellular entry and trafficking. Another plausible consideration is that cell membrane-derived LEs structurally rearrange lamellar domains, forming non-bilayer structural intermediates that promote nanoliposome cell interactions. Regardless of the exact mechanism(s) underlying the internalization of LE-modified nanoliposomes, first-line clinical agents like bortezomib warrant continued evaluation in diverse LE-modified nanosystems. 

RPMI 8226 LE-modified nanoliposomes preferentially targeted the source-originating RPMI 8226 cells, showing limited uptake by normal healthy cells and non-target cells of the BM (Figure 2). In contrast, LE-modified NCI-H929 nanoliposomes demonstrated a more generalized targeting profile; they interacted with both target and non-target BM cancer cells, though uptake by normal healthy BM cells remained limited (Figure 5). The specific criteria needed to select tumor tissue candidates to reliably produce LEs with high recognition potential remain unknown. For instance, lipid extracts derived from breast cancer cells preferentially target their own source cells [21]. RPMI 8226-LE exhibited similar characteristics. However, NCI H929-LE did not show this same source-specific targeting. That said, if a particular LE material type fails to show preferential source-cell interactions, the LE could still be highly advantageous if employed in the right clinical settings. It is therefore crucial not to rule out the value of materials that lack specific targeting capabilities until they are more thoroughly understood. Instead, future studies should build upon these benefits while systematically defining the limitations of utilizing established cell lines and cellular lipid extracts for clinical applications. Notably, nanosystems are constructed from commercially available ingredients derived from large-scale manufacturing. The procedures are well-established and reliably yield natural or synthetic-grade materials. However, nanosystems resulting from these processes frequently suffer from a lack of specificity, and often result in unwanted drug-associated side effects. That said, cellular membrane lipid extraction necessitates greater technical expertise and, given the nature of the cellular source material, could possibly require additional quality control measures for clinical translation. The deployment of cellular lipids could also pose other challenges regarding their production and manufacturing. 

It is possible that the use of established cell lines as the primary source material could fail to represent the diverse cell populations found in a real patient. Moreover, cell lines have adapted over years to survive in tissue culture flasks under controlled growth conditions. This may alter their gene expression and cellular lipid profile compared to malignant cells grown in humans, thus favoring patient-specific source material. Nonetheless, our data show that LE-modified nanoliposomes developed from established cell lines interact with cells more effectively than the unmodified, conventional variety. Consequently, further in vitro and in vivo validation is warranted to fully comprehend and maximize the therapeutic benefit of these LE materials.

## 4. Materials and Methods

### 4.1. Materials 

Chol (cholesterol), DOPC (1,2-dioleoyl-phosphatidylcholine), rhodamine-DPPE (1,2- dipalmitoyl-3-dipalmitoylphosphatidylethanolamine-N- (Lissamine rhodamine B sulfonyl)) were purchased from Avanti Polar Lipids (Alabaster, AL, USA). Nanoliposome sizes were achieved using a Branson 5800 multiple tube placement bath type sonicator purchased from Fisher Scientific (Hanover Park, IL, USA). FreeZone- Freeze Dry system was purchased from Labconco (Kansas City, MO, USA). Doxorubicin hydrochloride, gemcitabine hydrochloride, and carboplatin were purchased from Sigma Aldrich (St. Louis, MO, USA). Phosphate-buffered saline without calcium or magnesium and fetal bovine serum were purchased from Fisher Scientific (Hanover Park, IL, USA). Multiple myeloma (RPMI 8226), lymphoma (U937), and retinoblastoma (Y79) cell lines were purchased from ATCC (American Type Cell Collections, Manassas, VA, USA). Human PBMCs (peripheral blood mononuclear cells) were purchased from HumanCells Biosciences (Milpitas, CA, USA). Each vial of PBMCs contains dendritic cells, lymphocytes (T, B, and NK cells), and monocytes isolated from healthy donors. All cell lines were maintained in a NAPCO automatic CO_2_ cell culture incubator (Winchester, VA, USA) with 5% CO_2_ set at 37 °C. Live cell counts were determined by hemocytometer and Quadcount automated cell counter systems that quantified the fraction of live, dead, and total cell numbers in precise mode (Stellar Scientific, Baltimore, MD, USA). Dialysis for drug incorporation studies was performed using a Slide-A-Lyzer Dialysis Cassette G2- 10,000 MWCO (3 mL capacity) purchased from Thermo Scientific (Rockford, IL, USA). Max Q shaker- model 4450 was purchased from Thermo Scientific (Rockford, IL, USA). Spin-X Centrifuge Tube Filters (0.22 µm cellulose acetate in 2.0 mL Polypropylene) were employed to sterilize nanoliposomes by filtration and were purchased from Corning Incorporated (Salt Lake City, UT, USA). The fast flow low protein binding Millipore Express PLUS 0.22 µm -Stericup quick release systems used for bulk liquid sterilization were purchased from Fisher Scientific (Hanover Park, IL, USA). The CyQUANT™ LDH Cytotoxicity Assay kit (Invitrogen, Carlsbad, CA, USA) was employed to determine extracellular LDH levels.

### 4.2. Preparation of Nanoliposomes

Nanoliposomes consisted of DOPC, cholesterol, and/or lipid extract (LE) material derived from RPMI 8226 or NCI H929 cells. Components were mixed in the appropriate ratios. DOPC was included in preparations at the expense of LE or cholesterol content (typically ranging from 0 to 20 mol%). For studies involving fluorescence detection, 1 to 2 mol% DPPE-rhodamine lipid was typically included in the nanoliposome preparations. The concentration of total lipid employed, including LE material, depended on the experiment and was typically between 10 and 20 µmol/mL with or without drug content. Nanoliposomes were prepared by the thin-film method using a Buchi Rotovapor R-200 (Buchi Labortechnik AG, Flawil, Switzerland). The lipid film was hydrated with warm 1X PBS in an inert atmosphere, incubated at 37 °C, and vortexed intermittently between 5-min ice bath and warm water bath incubation periods. Nanoliposomes were sonicated using an Ultrasonic Bath- Branson model 5800 purchased from Fisher Scientific (Hanover Park, IL, USA). Sonication times varied as a function of the components in preparations (typically between 30 and 60 min). Nanoliposome size and zeta potential were measured at 25 °C in distilled water using a 90PLUS particle size and zeta potential analyzer (Brookhaven Instruments, Holtsville, NY, USA), and each value represents the mean ± SD of multiple different preparations. 

An amount of 3 or 5 mol% of chemotherapeutic agents was incorporated into each nanoliposome variety. Once the formulations were prepared, the free drug was separated from the drug-incorporated nanoliposomes by centrifuging the preparations at 10,000 rpm for 10 min at 25 °C. When dialysis was performed, the dialysis cassette G2 (MWCO 10,000) systems were employed [21]. Cassettes loaded with drug-loaded nanoliposomal preparations were placed in a 2-L glass beaker containing PBS buffer. Dialysis occurred overnight in a walk-in cold room in the absence of light. Particle size and zeta potential measurements were acquired. When sterile preparations were required, nanoliposomes were passed through Costar Spin-X centrifuge tube filters (membrane size—0.22 µm) purchased from Fisher Scientific (Hanover Park, IL, USA). 

### 4.3. Lipid Membrane Extraction Process

Bligh and Dyer introduced a rapid method for isolating lipids in frozen fish [36]. The method was later modified for the extraction of total tumor lipid membranes [21]. Briefly, target (RPMI 8226, NCI H929) cell lines were expanded in tissue culture flasks until cells reached about 80% confluency. Overcrowded cell populations and cells living in nutrient-deprived cultures were avoided. Next, three solvents were applied stepwise as follows: 1:2 chloroform–methanol mixture, chloroform, and distilled water, and vortexed intermittently between solvent additions. To obtain a two-phased system, the final mixture was centrifuged at 1000 rpm at 4 °C for 5 min. The bottom lipid layer was removed, transferred into a glass pyrex tube, and stored in a −80 °C freezer, with the process repeated until a sufficient total volume was obtained. A sufficient volume of extracted lipids was dehydrated to form a film using a rotary evaporator system. The heating bath Buchi B-491 rotary evaporator (Flawil, Switzerland) was set between 40 and 50 °C. Alharbi and Campbell [21] previously described the lyophilization and subsequent processing of the LE material.

### 4.4. Nanoliposome Binding and Cellular Uptake Studies

Cell suspensions were seeded ~1 × 10^4^ cells per ml of growth medium. Cells were placed in sterile 15 mL Bio-Reaction vented cap centrifuge tubes purchased from Fisher Scientific (Hanover Park, IL, USA). An amount of 100 nmoles of DPPE-rhodamine-labeled DOPC (100%) or DOPC/LE (95/5) nanoliposomal preparations was added to the respective tubes per ml of cell suspension. Centrifuge tubes were placed in a Max Q Shaker set at 37 °C for incubation periods between 0 and 60 min. The centrifuge tubes assigned to the 0-min time points were not placed in the shaker. To avoid cell settling and to promote maximum cellular-nanoliposome interactions for all groups, the shaker was set at a low and steady oscillation rate. Regardless of the sample type and time point monitored, all samples were vortexed slowly for a total of 5 s prior to placement in the Max Q shaker. The standardized practice was employed to distribute cell/nanoliposomes uniformly prior to the oscillation/incubation step. All samples were protected from direct light. Following removal of centrifuge tubes from the shaker, the tubes were placed on ice for 5 min prior to removing the unbound fraction of fluorescently labeled nanoliposomes by 1 to 2 centrifugation wash steps; the number of wash steps (with 1X PBS) remained consistent for a given experiment. Preparations consisting of the cell nanoliposome bound fraction were vortexed to achieve uniform cell suspensions. The final cell suspension was transferred to a 48-well plate for detection using a fluorescence microplate reader. Fluorescence intensity (A.U.) values were used to assess the relative cell binding/uptake.

### 4.5. LDH Cytotoxicity Assay Protocol 

*Kit preparation*: The contents of the LDH cytotoxicity assay kit were warmed to room temperature prior to use. Contents included the lysis and assay buffers and the stop and substrate solutions. An amount of 11.4 mL of diH_2_0 was added to the substrate mix. Next, in the absence of direct light, an amount of 600 µL of the assay buffer stock was mixed with the substrate stock solution.

*Cytotoxicity assay preparation*: An estimated 1 × 10^4^ multiple myeloma target (RPMI 8226), retinoblastoma control (Y79), or healthy peripheral blood mononuclear cells (PBMCs) was seeded in a 48-well plate per ml of growth medium, and subsequently placed in a cell culture type incubator set at 5% CO_2_ and 37 °C. Next, nanoliposomes loaded with drugs were added to the respective wells in a dose-dependent manner, and the well plate was placed in an incubator for 24 h. To determine the percent cytotoxicity, 50 µL of the treated cell suspension was next transferred to a 96-well plate, and an additional 50 µL of reaction mixture was next added to each well. Following 30-min incubation in the dark, 50 µL of the stop solution was next added, with an incubation period of 2 h. The absorbance was determined at 490 and 680 nm using a microplate reader. To prevent the pH indicator of phenol red from producing background absorbance overlapping with the 490 nm wavelength, phenol red-free medium was used for the LDH assay. Compound treated cytotoxicity was determined using the following formula:

$$\%\;Cytotoxicity\;=\;(\frac{Compound\;treated\;LDH\;activity\;-\;Spontaneous\;LDH\;activity}{Maximum\;LDH\;activity\;-\;Spontaneous\;LDH\;activity})\;\times \;100\;$$

### 4.6. Sulforhodamine B Assay Protocol

The suspension cells were seeded at 2 × 10^4^ cells per ml of growth medium in 24-well plates. Following 48 h of cell exposure to drug-loaded nanoliposomes in cell culture incubators, viable suspension cells were separated from non-viable cells by centrifugation. The cell pellet was resuspended in PBS and incubated with sulforhodamine B (SRB) reagent, followed by PBS washing steps to remove unbound SRB dye. Fluorescence intensity values were acquired at excitation and emission wavelengths of 555 nm and 590 nm, respectively [21].

### 4.7. In Vitro Drug Release Studies

Each drug agent was incorporated into either 100% DOPC or a DOPC/LE 95/5 or 90/10 binary mixture at a 3 mol% drug-to-lipid ratio. The two lipid extract materials employed in this study were derived from the RPMI 8226 and NCI H929 human MM cell lines. To resemble the conditions employed for the drug targeting and cytotoxicity studies reported here, each formulation was incubated in RPMI 1640 growth medium (w/o phenol red) within a humidified environment maintained at 37 °C and 5% CO_2_. The percentage of drug released from the nanoliposome preparations was quantified at predetermined time points (24 and 72 h) following the separation of the free drug from the incorporated drug. The release profile of doxorubicin was determined by measuring fluorescence intensity at excitation and emission wavelengths of 550 nm and 590 nm, respectively. UV spectrophotometry (absorbance wavelength of 270 nm) was used to detect bortezomib.

### 4.8. Statistical Analysis

Two-group comparisons were evaluated using Student’s *t*-test, whereas multiple-group comparisons were analyzed via one-way analysis of variance (ANOVA). Statistical significance is denoted as follows: * *p* < 0.05, ** *p* < 0.01, *** *p* < 0.001, and **** *p* < 0.0001.

## 5. Conclusions

In conclusion, this study supports the future development of cellular lipid-extracted nanoliposomes (CLENs) for enhancing drug delivery to multiple myeloma. Our findings demonstrate that cellular lipids derived from multiple myeloma cells can enhance both direct targeting and drug efficacy against multiple myeloma in vitro. For instance, RPMI 8226 LE selectively enhanced nanoliposome uptake exclusively by its source cell line. Conversely, other materials like NCI H929 LE functioned across different bone marrow cell types, but not in cell lines from other anatomical locations, such as retinoblastoma. The observations collectively suggest that the general tissue environment may also play a critical role in CLEN behavior. The additional integration of functional components, such as polyethylene glycol (PEG) and antibody conjugates, could potentially offer a range of other benefits. The capacity of these supplementary components to optimize physicochemical properties (e.g., molecular charge, zeta potential, drug release), or bolster targeting efficacy merits consideration. Future studies should clarify how LE-modified nanoliposomes enter cells and compare LEs derived from established tumor cell lines with those from patient-derived materials.

## Figures and Tables

**Table 1:** Formulation and cellular optimization for RPMI-8226 CLENs. * *p* values reflect diff. in cellular uptake between groups.

Item No.	Composition	Percent-Ratio	Size (nm)	Cell Uptake FI (A.U.)	Item Analysis	Difference FI (A.U.)	* *p* Value
(1)	DOPC	100%	183 ± 10	20,381	--	--	--
(2)	DOPC/Chol	95/5	257 ± 8	25,808	1 and 2	5427	*p* < 0.001
(3)	DOPC/LE	95/5	244 ± 11	29,008	1 and 3	8627	*p* < 0.001
(4)	DOPC/Chol/LE	90/5/5	311 ± 5	26,572	1 and 4	6191	*p* < 0.001
	--	--	--	--	2 and 4	763	*p* < 0.05
	--	--	--	--	3 and 4	2436	*p* < 0.001

**Table 2:** Overall formulation results of various cytotoxic drug-loaded RPMI-8226 nanoliposomes.

Composition	Percent-Ratio	Drug Incorporated (3 mol%)	Size (nm) (*n* = 4)	Zeta Potential (mV) (*n* = 4)
DOPC	100%	--	183 ± 10	0.70 ± 4
DOPC/LE	95/5	--	244 ± 1	−0.93 ± 3
DOPC	100%	Doxorubicin	224 ± 7	0.53 ± 4
DOPC/LE	95/5	Doxorubicin	266 ± 3	−0.89 ± 5
DOPC	100%	Carboplatin	88 ± 9	0.83 ± 5
DOPC/LE	95/5	Carboplatin	111 ± 6	−2.34 ± 6
DOPC	100%	Gemcitabine	112 ± 2	1.34 ± 4
DOPC/LE	95/5	Gemcitabine	98 ± 2	−1.12 ± 6

**Table 3:** Drug release characteristics of LE-modified nanoliposomal formulations.

Composition	Lipid Ratio/Percent	%EE (*n* = 4)	24 h (*n* = 4)	72 h (*n* = 4)
DOPC + Dox	100%	90 ± 4	1.3%	2.6%
DOPC/RPMI 8226-LE + Dox	95:5	93 ± 2	1.4%	3.1%
DOPC/NCI H929-LE + Dox	90:10	91 ± 4	1.2%	8.2%
DOPC + BTZ	100%	98 ± 2	3.3%	6.9%
DOPC/RPMI 8226-LE + BTZ	95:5	95 ± 1	4.9%	4.3%
DOPC/NCI H929-LE + BTZ	90:10	96 ± 3	1.9%	5.2%

## Data Availability

The datasets generated during this study are available from the corresponding author on reasonable request.

## Abbreviations

The following abbreviations are used in this manuscript:

BM	Bone marrow
Chol	Cholesterol
DOPC	Dioleoyl-phosphatidyl-choline
FBS	Fetal bovine serum
K562-GFP	Chroni myeloid leukemia cell line-human
LDH	Lactate dehydrogenase
LE	Lipid extract
MM	Multiple myeloma
NCI H929	Multiple myeloma cell line-human
PBMCs	Peripheral blood mononuclear cells-human
PDI	Polydispersity index
RPMI 1640	Roswell Park Memorial Institute w/o phenol red
RPMI 8226	Multiple myeloma cell line-human
SEER	Surveillance Epidemiology and End Results
U937	Lymphoma cell line-human
Y79	Retinoblastoma cell line-human
